# Modified Huangqi Chifeng Decoction Attenuates Proteinuria by Reducing Podocyte Injury in a Rat Model of Immunoglobulin a Nephropathy

**DOI:** 10.3389/fphar.2021.714584

**Published:** 2021-07-26

**Authors:** Meiying Chang, Bin Yang, Liusheng Li, Yuan Si, Mingming Zhao, Wei Hao, Jinning Zhao, Yu Zhang

**Affiliations:** ^1^ Department of Nephrology, Xiyuan Hospital, China Academy of Chinese Medical Sciences, Beijing, China; ^2^ Department of Pathology, Xiyuan Hospital, China Academy of Chinese Medical Sciences, Beijing, China; ^3^ Medical Animal Experimental Center, Xiyuan Hospital, China Academy of Chinese Medical Sciences, Beijing, China

**Keywords:** modified huangqi chifeng decoction, IgA nephropathy, podocyte injury, nephrin, podocalyxin

## Abstract

Modified Huangqi Chifeng decoction (MHCD) has been used to reduce proteinuria in immunoglobulin A nephropathy (IgAN) for many years. Previously, we have demonstrated its protective role in glomerular mesangial cells. Podocyte injury, another key factor associated with proteinuria in IgAN, has also attracted increasing attention. However, whether MHCD can reduce proteinuria by protecting podocytes remains unclear. The present study aimed to investigate the protective effects of MHCD against podocyte injury in a rat model of IgAN. To establish the IgAN model, rats were administered bovine serum albumin, carbon tetrachloride, and lipopolysaccharide. MHCD in three doses or telmisartan was administered once daily for 8 weeks (*n* = 10 rats/group). Rats with IgAN developed proteinuria at week 6, which worsened over time until drug intervention. After drug intervention, MHCD reduced proteinuria and had no effect on liver and kidney function. Furthermore, MHCD alleviated renal pathological lesions, hyperplasia of mesangial cells, mesangial matrix expansion, and podocyte foot process fusion. Western blot analysis revealed that MHCD increased the expression of the podocyte-associated proteins nephrin and podocalyxin. Additionally, we stained podocyte nuclei with an antibody for Wilms’ tumor protein one and found that MHCD increased the podocyte number in rats with IgAN. In conclusion, these results demonstrate that MHCD attenuates proteinuria by reducing podocyte injury.

## Introduction

Immunoglobulin A (IgA) nephropathy (IgAN) is a glomerular disease characterized by IgA deposition in the mesangial region. Its clinical manifestations include proteinuria, hematuria, acute kidney injury, hypertriglyceridemia, and hypertension. IgAN is the main cause of end-stage renal disease in patients with primary glomerular disease in China (Yang et al., 2020). Although clinical manifestations vary, the majority of patients with IgAN exhibit progressive symptoms, with progression to end-stage renal disease within 10 years after diagnosis in 10–30% of cases (Liu et al., 2018). While progression is driven by podocyte injury and depletion (Hishiki et al., 2001; Lemley et al., 2002; Tomino, 2007; Xu et al., 2010), podocyte injury alone is the dominant cause (Menon et al., 2013; Liu et al., 2018). Therefore, the mechanism by which podocyte injury contributes to IgAN warrants investigation and might provide a basis for therapeutic strategies to delay progression.

Glomerular podocytes, highly specialized epithelial cells, together with the glomerular basement membrane, play an important role in glomerular filtration (Asao et al., 2012). Podocytes stabilize the glomerular architecture against the distention of the glomerular basement membrane by providing a large filtration surface through the slit diaphragm structure. In addition to the crucial interaction between podocyte foot extensions controlling glomerular filtration, certain proteins (i.e., nephrin, podocin, and podocalyxin) involved in the maintenance of the structural and functional integrity of the filtration barrier in the kidney have been identified (Mundel and Shankland, 2002; Asao et al., 2012; Akankwasa et al., 2018). Podocalyxin is present on the apical aspects of podocytes and offers structural support to the capillary loop, and nephrin is expressed on the lateral aspect extending into the slit diaphragm and impedes the filtration of large molecules into the urinary space (Akankwasa et al., 2018). Changes in the expression and distribution of nephrin precede and can cause foot process fusion and proteinuria (Lu et al., 2013). The role of podocytes in IgAN requires further investigation.

Several traditional Chinese medicines have been used for the treatment of IgAN, and their effects have been ascertained by clinical and animal experiments (Yang et al., 2018; Bai et al., 2019a; Deng et al., 2019; Li et al., 2020a; Li et al., 2020b). Modified Huangqi Chifeng decoction (MHCD), a compound formula in traditional Chinese medicine, comprises seven herbs: *Astragalus membranaceus* Bge, *Euryale ferox* Salish, *Rosae Laevigatae* Fructus, *Radix Paeoniae* Rubra, *Saposhnikoviae* Radix, *Rhizoma Dioscoreae* Nipponicae, *Hedyotis Diffusae* Herba. MHCD has been shown to alleviate proteinuria in patients with IgAN in single-case-control and real-world studies (Jiao and Zhang, 2018; Yu and Zhang, 2018). Furthermore, MHCD protects against renal fibrosis and podocyte injury in a rat model induced by doxorubicin or in mesangial cells induced by lipopolysaccharide (LPS) (Gao et al., 2016; Jiao and Li, 2016; Liu and Li, 2016; Yu et al., 2018). However, only a few studies have focused on podocyte protection using traditional Chinese medicine in rats with IgAN. In this study, we investigated the protective effects of MHCD on podocytes in rats with IgAN, and analyzed the molecular and cellular mechanisms underlying these effects.

## Materials and Methods

### Chemicals and Reagents

MHCD comprises seven herbs: 30 g Sheng Huangqi (Astragalus membranaceus Bge), 20 g Qian Shi (Euryale ferox Salish), 10 g Jin Ying-zi (Rosae Laevigatae Fructus), 10 g Chi Shao (Radix Paeoniae Rubra), 10 g Fang Feng (Saposhnikoviae Radix), 20 g Chuan Shan-long (Rhizoma Dioscoreae Nipponicae), and 20 g Bai Hua-she-she-cao (Hedyotis Diffusae Herba). The herbs were purchased from the Xiyuan Hospital of the China Academy of Chinese Medical Sciences. The herbs were initially soaked in water for 1 h and then boiled twice for 30 min each time. The liquid was sealed, vacuum dried, and stored in a glass bottle in a refrigerator at 4°C until use. Telmisartan (Micardis; 80 mg/pill) was purchased from Boehringer Ingelheim International GmbH (Ingelheim am Rhein, Germany). Bovine serum albumin (BSA), LPS, and carbon tetrachloride (CCl4) were purchased from Sigma-Aldrich (St. Louis, MO, United States).

The primary antibodies and Alexa Fluor 488-labeled goat anti-rabbit IgG secondary antibodies (No. ZA-0446 and ZF-0511, respectively) for immunofluorescence assays were purchased from ZSGB Biotechnology Co., Ltd. (Beijing, China). The periodic acid Schiff (PAS) Stain Kit was purchased from X-Y Biotechnology (No. XY7640; Shanghai, China). The Masson’s Trichrome Stain Kit was purchased from Solarbio (Beijing, China). The primary antibody for Wilms’ tumor protein 1 (WT1) was purchased from Novus (No. NBP2-44606; St. Charles, MO, United States). The anti-mouse/rabbit IgG secondary antibodies were purchased from ZSGB-BIO (No. PV-6000; Beijing, China). The primary antibodies for nephrin and podocalyxin (No. A3048 and A10200, respectively) for western blot analysis were obtained from Abclonal Technology (Wuhan, China). Goat anti-rabbit IgG-HRP was obtained from Jackson ImmunoResearch (No. 111-035-003; West Grove, PA, United States). RIPA lysis buffer and the BCA Assay Kit (No. R0010 and PC0020, respectively) were purchased from Solarbio.

### Animal Groups and Treatments

Sixty-six 3–5-week-old male Sprague–Dawley rats, weighing 150 ± 10 g, were purchased from Beijing Vital River Laboratory Animal Technology Co., Ltd. (Beijing, China). Animal welfare and experimental procedures were performed in strict accordance with the guidelines of the Animal Ethics Committee of Xiyuan Hospital of China Academy of Chinese Medical Sciences. Animals were housed in humidity-controlled rooms (60 ± 10%) at 24 ± 1°C with a 12 h light/dark cycle and free access to standard food and tap water. All rats were housed in metabolic cages and acclimated to laboratory conditions for 7 days, after which they were randomly divided into either the blank (*n* = 13) or model (*n* = 53) group.

**FIGURE 1:**
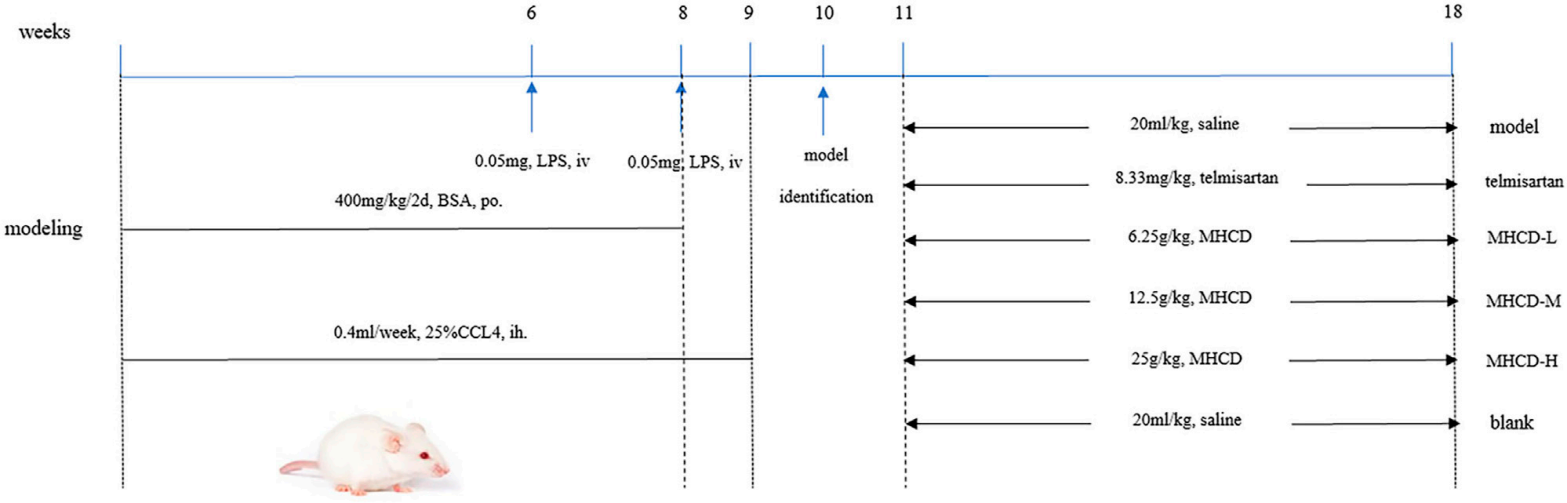
Protocol of IgAN rat modeling and treatment. After oral administration of BSA for 8 weeks, hypodermic injection of CCl4 for 9 weeks and two times of LPS with tail intravenous injection, the IgAN rats were orally administered different drugs for 8 weeks.

The rat IgAN model was established by administering BSA, LPS, and CCl4 according to previously described methods (Peng et al., 2013; Zhang et al., 2014; Liu et al., 2018), with slight modifications (Figure 1). In brief, the immunogen BSA was intragastrically administered at 400 mg/kg once every 2 days for eight consecutive weeks, and 0.1 ml CCl4 dissolved in 0.3 ml castor oil was subcutaneously administered weekly for 9 weeks. During weeks 6 and 8, LPS (0.05 mg) was injected through the tail vein. Rats in the blank group were intragastrically administered an equal volume of distilled water and were administered equal amounts of physiological saline through tail vein injection (to match the LPS injection) and via the subcutaneous route (CCl_4_). The methods for administration were the same as those used in the model group. At week 10, three rats from both the blank and model groups were used for model validation. After verifying successful model establishment, the blank group was maintained (*n* = 10), and rats in the model group (*n* = 50) were randomly divided into the following five groups: model group, telmisartan group, MHCD-H group, MHCD-M group, and MHCD-L group (*n* = 10 rats/group). The corresponding drug intervention for each group was initiated at week 11. Rats in the telmisartan group were intragastrically administered telmisartan at a dose of 8.33 mg/kg/d. Rats in the MHCD-H, MHCD-M, and MHCD-L groups were intragastrically administered MHCD at 25, 12.5, and 6.25 g/kg/d, respectively, for 8 weeks until the end of week 18. The blank and model groups received equal volumes of normal saline. All drugs were diluted with distilled water, and the dosages were evaluated by body surface coefficient conversion between humans and rats.

### Sample Collection and Preparation

Urine was collected from rats in metabolic cages to determine 24 h proteinuria levels. Urine was collected once every 2 weeks. After the last intragastric administration at week 18, the rats were anesthetized with 3% chloral hydrate and the blood was obtained from the abdominal aorta to determine the levels of albumin, serum creatinine, blood urea nitrogen, alanine transaminase, and aspartate transaminase, using an automatic biochemical analyzer. After blood was drawn from the abdominal aorta, the kidney was rapidly harvested. A part of the renal cortex was placed in 10% formalin fixative solution for optical microscopy and immunohistochemical detection, a portion was fixed in 20% glutaraldehyde solution for electron microscopy and subsequent immunofluorescence detection, and a portion was stored at −70°C for western blot analysis.

### Immunofluorescence Intensity of Immunoglobulin A

As demonstrated in Table 1, immunofluorescence intensity was determined using the standard five-point semi-quantitative method. At least ten glomeruli (magnification, ×400) were observed in each section. They were scored according to the intensity of microscopic expression in each glomerulus.

**TABLE 1:** Semiquantitative standard for IgA deposition.

Immunofluorescence deposition under light microscope	Immunofluorescence intensity of IgA
No fluorescence under low-power lens but seemingly visible under high-power lens	—
Fluorescence appears to be visible under low-power lens and can be seen with high-power lens	＋
Fluorescence can be seen under low-power lens and can be clearly seen under high-power lens	＋＋
Fluorescence can be clearly seen under low-power lens and is strong under high-power lens	＋＋＋
Fluorescence is strong under low-power lens and very strong under high-power lens	＋＋＋＋

### Histopathological Analysis

Sections of cortical tissues were fixed in 10% buffered formalin at room temperature for 48 h, embedded in paraffin, and sliced to 4 µm-thick sections. The sections were stained at room temperature with hematoxylin-eosin (HE) for 3 min, followed by hydrochloric acid and alcohol for differentiation, and a graded series of alcohol and xylene dehydration. Sections were sealed, and PAS and Masson’s trichrome staining were performed according to the kit instructions. Photomicrographs of HE-, PAS-, and Masson’s trichrome-stained sections were obtained under a light microscope (magnification, ×200).

### Immunohistochemistry Assay

Podocyte counts can be obtained based on the expression of WT1 in glomeruli. The paraffin sections were first dewaxed with xylene and a graded series of alcohol. After using sodium citrate for antigen retrieval, endogenous peroxidase was quenched with 3% hydrogen peroxide, and the sections were incubated with the WT1 antibody (1:100) overnight at 4°C. The sections were incubated with PV-6000 for 30 min at room temperature, followed by hematoxylin redyeing, gradient alcohol dehydration, and neutral gum sealing. Finally, under a light microscope, brown-yellow particles in the nucleus indicated positive expression. Five non-overlapping fields were randomly observed under a microscope (magnification, ×400); positive cells in each field were counted with Image-Pro Plus 6.0 analysis software, and the average value was obtained.

### Electron Microscopy

Renal cortical tissue was fixed in 2.5% glutaraldehyde at 4°C for 24 h, washed with phosphate-buffered saline, and sliced to a thickness of 70–90 nm. After staining with osmium tetroxide and lead citrate at room temperature for 5–10 min, changes in the podocyte foot process and its fine structure were observed under a Hitachi H-600 transmission electron microscope (magnification, ×8,200 and ×16,500; Hitachi, Ltd., Tokyo, Japan).

### Western Blot Analysis

Total protein was extracted from approximately 30 g of kidney tissue with RIPA buffer containing PMSF. The protein concentration was measured using a BCA kit. Proteins were separated using 8–10% gradient SDS-PAGE and then transferred to a PVDF membrane. The membrane was then incubated with primary antibodies against nephrin (1:2000) and podocalyxin (1:2000) overnight at 4°C after blocking with TBST containing 5% skim milk. After incubation with secondary antibodies for 1 h, membranes were treated with ECL chemiluminescence reagents. ImageJ 4.0 was used to analyze the grayscale values for each group, and β-actin was used as the internal reference protein.

### Statistical Analysis

All values are expressed as means ± standard deviation (SD). One-way ANOVA for multiple comparisons was used to analyze differences among groups. The Kruskal–Wallis test was used for comparisons of fluorescence intensity data. Statistical analyses were performed using GraphPad Prism (version 8.0) and SPSS (version 20.0). The threshold for statistical significance was set at *p* < 0.05.

## Results

### General Condition of Rats in Each Group

After each subcutaneous injection of CCl_4_, rats in the model group exhibited reduced activity and listlessness, with gradual recovery beginning the next day. After the injection of LPS during week 6, rats in the model group exhibited a reduction in food intake, messy hair, and listlessness, which typically returned to normal in approximately 6 days. Rats in the blank group exhibited free movement, a normal diet, and smooth body hair.

### Modified Huangqi Chifeng Decoction Ameliorates Proteinuria

**FIGURE 2:**
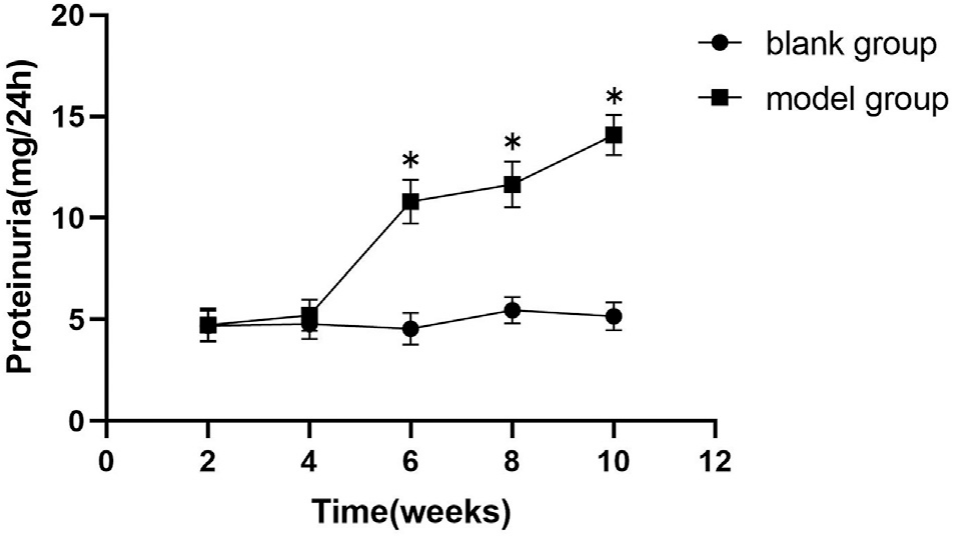
The 24 h proteinuria of the model group rats increases significantly from the week 6 of the experiment. Data are expressed as means ± standard deviation (SD). *p < 0.01 vs. blank group.

**FIGURE 3:**
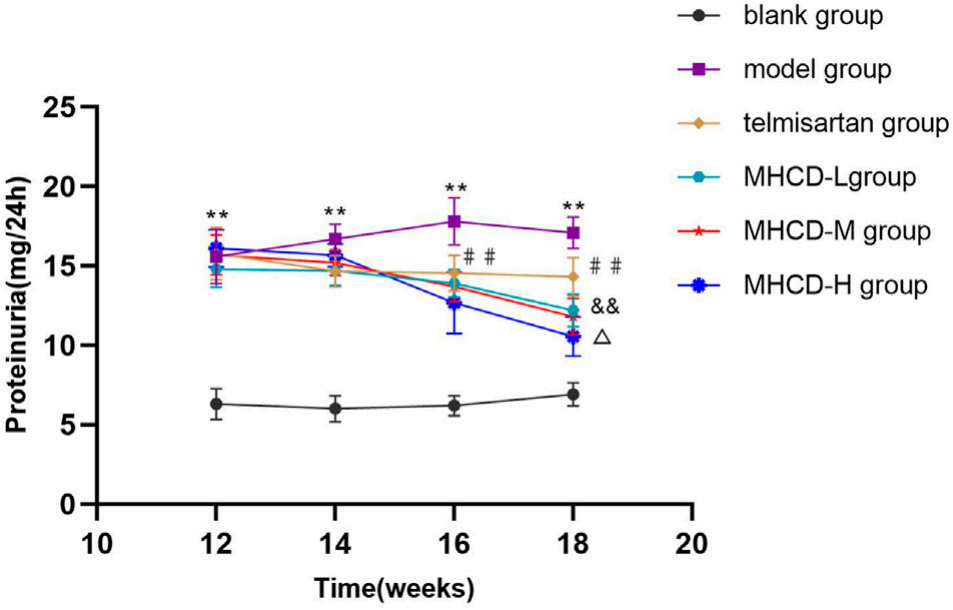
MHCD ameliorates proteinuria over time. Data are expressed as means ± SD (*n* = 10). **p < 0.01 vs. blank group;＃＃*p* < 0.01 vs. model group; && *p* < 0.01 vs. Telmisartan group;△ *p* < 0.05 vs. MHCD-L group. MHCD, modified Huangqi Chifeng decoction.

As demonstrated in Figure 2 and Figure 3, proteinuria in the model group began to increase at week six and worsened over time until drug intervention. After drug intervention, 24 h proteinuria was lower in rats in the telmisartan and MHCD groups than in those in the model group (both *p* < 0.01). Compared to the telmisartan group, all MHCD groups showed reduced 24 h proteinuria (all *p* < 0.01). Rats in the MHCD-H group showed significantly lower 24 h proteinuria than those in the MHCD-L group (*p* < 0.05), indicating that MHCD ameliorates proteinuria in rats with IgAN.

### Modified Huangqi Chifeng Decoction Does Not Alter Certain Biochemical Measurements of Liver and Kidney Function

**FIGURE 4:**
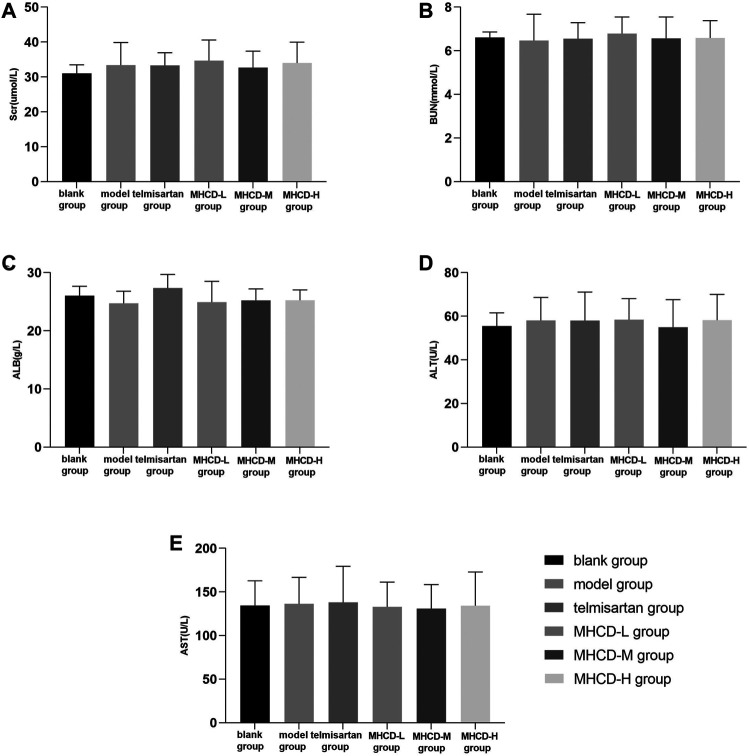
MHCD does not alter certain biochemical measurements of liver and kidney function while ameliorating proteinuria. Data are expressed as means ± SD (*n* = 10). Scr, serum creatinine; BUN, blood urea nitrogen; ALB, albumin; ALT, alanine transaminase; AST, aspartate transaminase.

Besides efficacy, we evaluated the safety indexes in rats. The results showed no significant differences in five biochemical parameters between rats in the blank group and those in the other groups (all *p* > 0.05; Figure 4). These results further support the application of MHCD.

### Modified Huangqi Chifeng Decoction Reduces Immunoglobulin A Deposition

**FIGURE 5:**
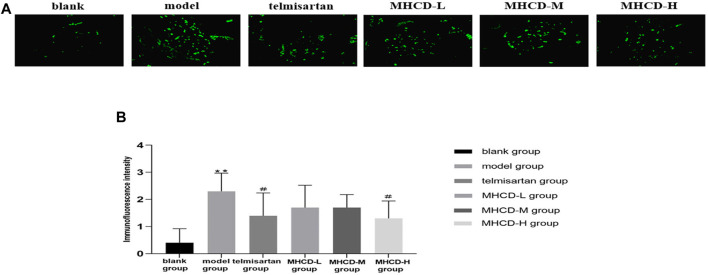
MHCD reduces IgA deposition. **(A)** The IgA immunofluorescence deposition in glomeruli of the six groups (magnification × 400). **(B)** MHCD ameliorates the immune injury. Data are expressed as means ± SD (*n* = 10). **p < 0.01 vs. blank group;＃*p* < 0.05 vs. model group.

IgA immunofluorescence was nearly undetectable in the glomeruli of rats in the blank group. In the model rats, IgA was strongly expressed in the glomerular mesangium. Rats in the telmisartan group showed lower IgA immunofluorescence than those in the model group (Figure 5A). After drug intervention, IgA deposition was significantly lower in rats in the telmisartan and MHCD-H groups than in those in the model group (both *p* < 0.05; Figure 5B). These results suggest that MHCD alleviates immune injury in rats with IgAN.

### Modified Huangqi Chifeng Decoction Ameliorates Renal Histopathology Injury

**FIGURE 6:**
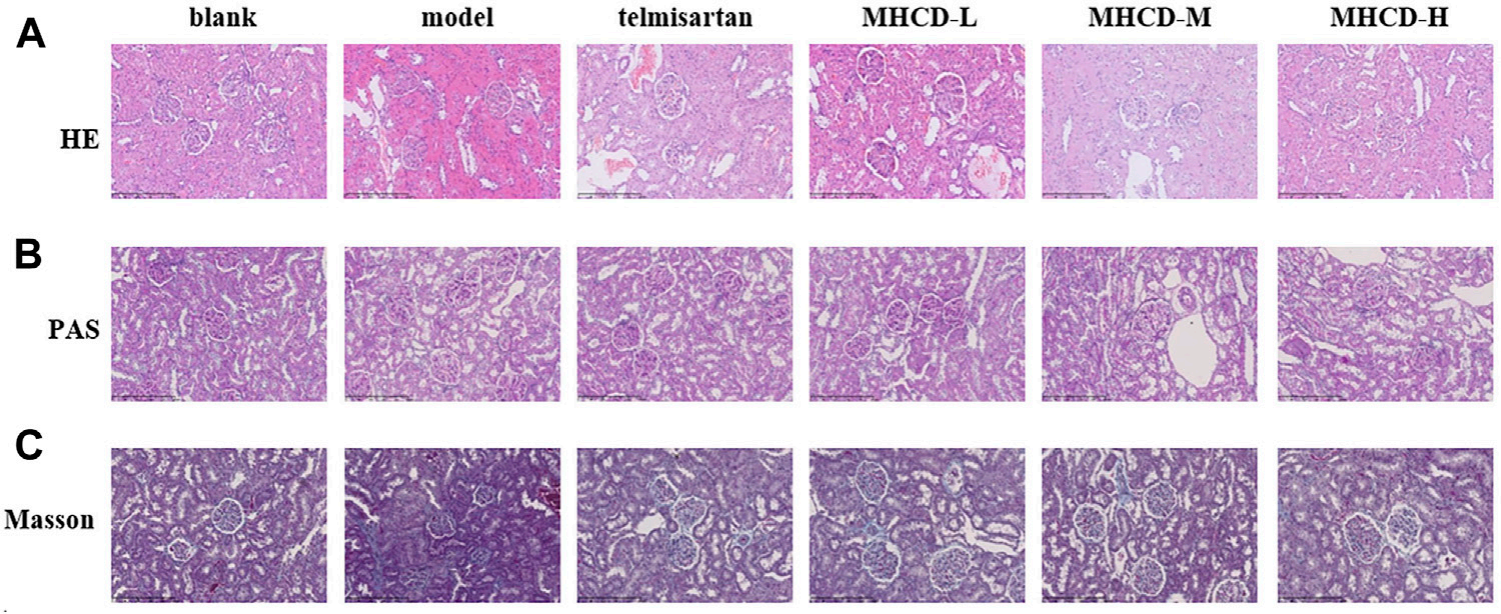
MHCD ameliorates renal histopathology injury **(A)** Pathologic changes with HE-stained in renal tissue of the six groups (magnification × 200). **(B)** Pathologic changes with PAS-stained in renal tissue of the six groups (magnification × 200). **(C)** Pathologic changes with Masson-stained in renal tissue of the six groups (magnification × 200).

Renal pathological changes were examined by HE, PAS, and Masson’s trichrome staining (Figure 6). Compared to the blank group, the model group had a greater number of proliferative mesangial cells, increased extracellular matrix deposition, a thickened glomerular basement membrane, and disordered tubular cells. Telmisartan and MHCD treatments ameliorated the renal pathological lesions in rats with IgAN. These results demonstrate the protective effect of MHCD on the kidneys of rats with IgAN.

**FIGURE 7:**
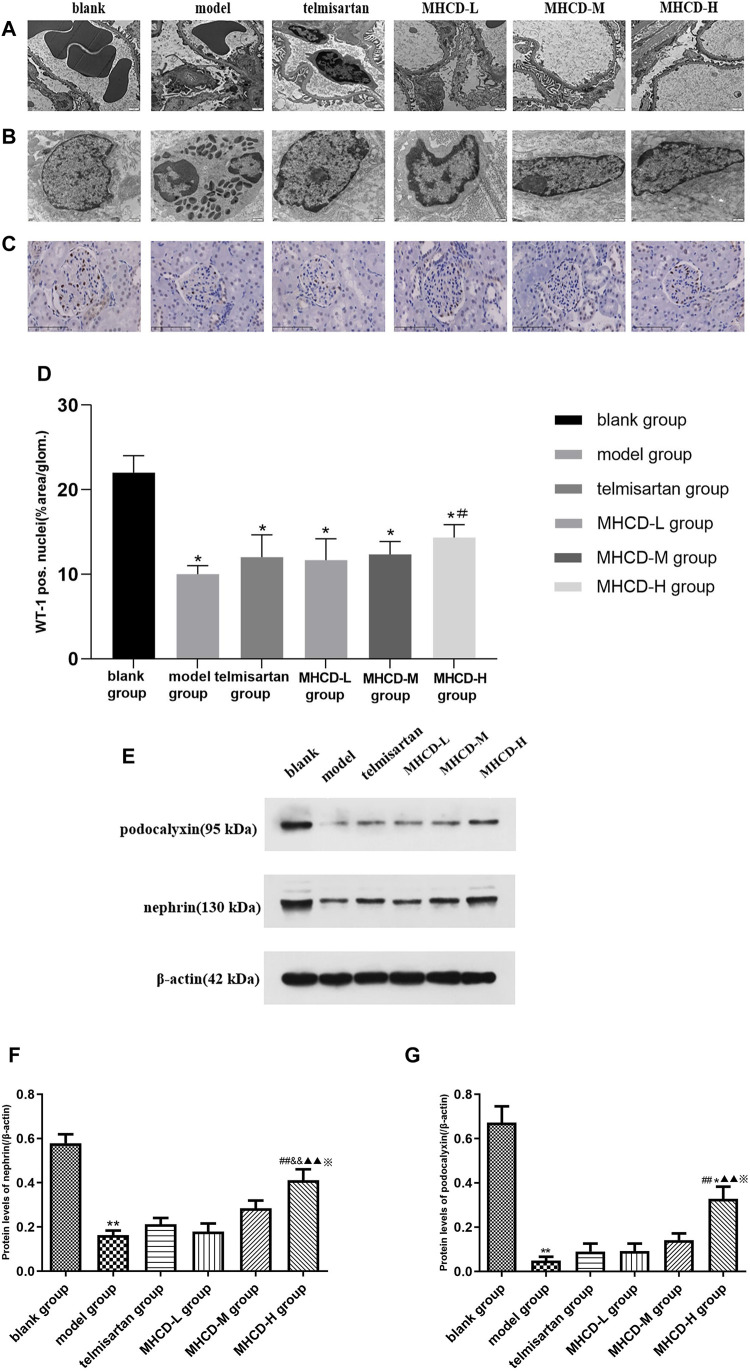
MHCD protects against podocyte injury. **(A)** The foot process and basement membrane were observed under electron microscopy (magnification × 8,200). **(B)** Changes in the fine structure of the foot process were observed under electron microscopy (magnification × 16,500). **(C)** WT1 immunohistochemistry of the six groups (magnification × 400). **(D)** Average percent staining area and WT1-positive podocytes per glomerulum in the IgAN rat model obtained using Image-Pro Plus 6.0 analysis software. Data are expressed as means ± SD (*n* = 3). *p < 0.05 vs. blank group;＃*p* < 0.05 vs. model group. **(E–G)** Protein levels of nephrin and podocalyxin in the six groups. For relative quantification of proteins, β-actin was used as an internal control. The relative expression of proteins was calculated using Image J. Data are expressed as means ± SD (*n* = 3). ***p < 0.01 vs. blank group;＃＃*p* < 0.01 vs. model group; &&*p* < 0.01 vs. Telmisartan group; ▲▲*p* < 0.01 vs. MHCD-L group; ※*p* < 0.05 vs. MHCD-M group; *p < 0.05 vs. telmisartan group.

As determined by electron microscopy (Figure 7A, ×8,200), the foot process and basement membrane of rats in the blank group exhibited a normal morphology; the podocyte foot processes were arranged in an orderly manner and were uniform in size. Podocytes in model group rats were flattened or fused, along with a thickened basement membrane and an increased number of proliferating mesangial cells. The degree of fusion of podocyte foot processes in rats in all MHCD groups was lower than that in those in the model group, to various extents.

### Modified Huangqi Chifeng Decoction Protects Against Podocyte Injury

Changes in the fine structure of the foot process were observed via electron microscopy (Figure 7B; ×16,500). The podocyte cell body in rats in the blank group was typical, with a high number of organelles, clear nucleoli, smooth and complete nuclear membrane edges, fine chromatin, and a uniform distribution in the nuclei. In model group rats, podocytes shrank substantially, intracytoplasmic organelles decreased, and chromatin agglomeration and nuclear fragmentation were detected. Atrophy of podocytes was reduced, and the number of intracytoplasmic organelles was higher in rats in the MHCD groups than in those in the model group.

WT1 immunohistochemistry was used to evaluate the morphological state of podocytes and to stain nuclei (Figure 7C). After drug intervention, the number of WT1-positive podocytes was significantly higher in rats in MHCD-H than in those in the model group (*p* < 0.05; Figure 7D). These results further support the protective effect of MHCD on the podocytes of rats with IgAN.

### Modified Huangqi Chifeng Decoction Increases the Expression of Podocyte-Associated Proteins

To further investigate the molecular mechanism by which MHCD affects podocytes in rats with IgAN, the levels of the podocyte-associated proteins nephrin and podocalyxin were evaluated. Nephrin levels were significantly lower in rats in the model group than in those in the blank group (*p* < 0.01), but were higher in rats in the MHCD-H group than in those in the model group (*p* < 0.01; Figures 7E, F). Furthermore, nephrin levels were significantly higher in rats in the MHCD-H group than in those in all other groups (all *p* < 0.05). Podocalyxin levels were also significantly lower in rats in the model group than in those in the blank group (*p* < 0.01), but were higher in rats in the MHCD-H group than in those in the model group (*p* < 0.01; Figures 7E, G). Podocalyxin levels were significantly higher in rats in the MHCD-H group than in those in all other groups (all *p* < 0.05).

## Discussion

IgA nephropathy is the most common glomerulonephritis worldwide. According to the 2012 KDIGO clinical practice guideline, we recommend long-term angiotensin-converting enzyme inhibitor (ACE-I) or angiotensin receptor blocker (ARB) treatment when proteinuria is > 1 g/d, with up-titration of the drug depending on blood pressure (Beck et al., 2013). It has been reported that angiotensin II (AngII) plays an important role in the injury of podocytes. AngII can lead to the reorganization of actin cytoskeleton and induction of podocyte apoptosis (Ren et al., 2012; Wang et al., 2016). In addition, some studies have confirmed the protective effect of telmisartan on podocytes (Villa et al., 2011; Fukami et al., 2013). Therefore, telmisartan, as a commonly used drug ARB, was selected as the positive control in this study. For patients with persistent proteinuria of ≥1 g/d, despite 3–6 months of optimized supportive care (including ACE-I or ARBs and blood pressure control), and GFR of >50 ml/min per 1.73 m^2^, according to the 2012 KDIGO clinical practice guideline, we recommend a 6-months course of corticosteroid therapy (Beck et al., 2013). However, the role of immunosuppression in the management of IgA nephropathy remains highly controversial. The STOP-IgAN and TESTING trials were terminated due to adverse events (Lv et al., 2017; O’Shaughnessy and Lafayette, 2017). Therefore, it is of social significance and scientific value to seek new treatment methods.

In China, traditional Chinese medicines (TCMs) have an extensive history of application, and there is substantial literature on the treatment of IgAN with TCM. Furthermore, various TCM extracts or formulae have been demonstrated to have renal-protective effect (Li et al., 2017; Yang et al., 2018; Bai et al., 2019a; Bai et al., 2019b; Li et al., 2020a; Li et al., 2020b; Li et al., 2020c; Li and Li, 2020). Modified Huangqi Chifeng decoction, a compound formula in TCM, has been used to treat IgAN for several years. The results of this study clearly demonstrate the protective effect of MHCD on glomerular podocytes.

Proteinuria is one of the main clinical symptoms of IgAN. As expected, rats in the model group exhibited a marked increase in proteinuria at the start of week 6, which gradually increased thereafter. Proteinuria in rats in the MHCD and telmisartan groups started to decline from week 14. Modified Huangqi Chifeng decoction, especially at a high dose, attenuated proteinuria and did not alter biochemical parameters of liver and kidney functions in rats.

Histological changes, including mesangial cell proliferation, increased mesangial matrix, tubulointerstitial inflammation, fibrosis, and podocyte fusion, were also observed in the present study, which is consistent with previous findings (Coppo, 2017; Coppo, 2019). After MHCD intervention, pathological damage to kidney tissues was relieved to various degrees, as evidenced by light microscopy, and the IgA deposition in MHCD-H-treated rats in the mesangial region of the glomeruli was also reduced, as evidenced by immunofluorescence microscopy. Under electron microscopy, compared to that in rats in the model group, the extent of podocyte foot process fusion in rats in all MHCD groups was reduced to various degrees, podocyte atrophy was alleviated, and the number of intracytoplasmic organelles was increased. In addition, MHCD increased the expression of the podocyte-associated proteins nephrin and podocalyxin and increased the number of podocytes in rats with IgAN.

Proteinuria is an early consequence of podocyte injury and a typical sign of kidney disease (Nagata, 2016). Endothelial cells of glomerular capillaries, the surrounding podocytes, and the fused extracellular matrix form the glomerular filtration barrier (Scott and Quaggin, 2015). Structurally, podocytes serve as the last gatekeepers in the glomerular filtration barrier. The foot processes of podocytes contain an actin-based cytoskeleton linked to the glomerular basement membrane (Reiser et al., 2000). The foot processes of podocytes form a highly-branched interdigitating network with the foot processes of neighboring podocytes. Slit diaphragm bridges the filtration slits between opposing podocyte foot processes, thereby forming the final barrier to urinary protein loss (Leung et al., 2018). Haraldsson *et al.* also pointed out that highly differentiated and specialized podocytes are of utmost importance for an intact barrier (Haraldsson et al., 2008). Podocyte foot processes are maintained by the actin cytoskeletal system, and nephrin and podocalyxin lead to loss of the foot process, which is closely associated with proteinuria (Nagata, 2016; Akankwasa et al., 2018).

Podocyte stress and adaptation observed in podocytopathies manifest as alterations in the podocyte charge or shape, an active process resulting from the rearrangement of the actin cytoskeleton preceding podocyturia (i.e., the shedding of viable podocytes in urine) (Carney, 2013; Kriz et al., 2013; Nagata, 2016). Podocyte injury is used as a clinical prognostic index for glomerular diseases (Asanuma and Mundel, 2003; Xu et al., 2010; Jiang et al., 2012; Chen et al., 2017). Lu *et al.* found that the expression of nephrin in rats with IgAN increases at an early timepoint and decreases thereafter, suggesting this increase to be a compensatory change and that the decrease in nephrin levels could be related to podocyte injury (Lu et al., 2013). Jiang *et al.* reported that the number of urinary podocytes reflects the loss of podocytes in renal tissues, which might be a marker of IgAN progression (Jiang et al., 2012). Indeed, podocyte injury precedes the increase in proteinuria; therefore, podocytes are primary therapeutic targets for IgAN (Xu et al., 2010).

In the present study, the levels of the podocyte-associated proteins podocalyxin and nephrin were reduced in rats with IgAN. MHCD augmented the levels of these proteins as well as the number of podocytes, as determined by WT1 staining. After drug intervention, the numbers of WT1-positive podocytes in rats in MHCD-H group increased significantly (*p* < 0.05). MHCD reduced podocyte foot process fusion, suggesting that it has an obvious protective effect on podocytes.

In conclusion, the results of the present study suggest that MHCD ameliorates proteinuria in rats with IgAN. Additionally, light microscopy, electron microscopy, and western blot analysis demonstrated that MHCD exerts a therapeutic effect on IgAN *in vivo* by maintaining podocyte function. Thus, MHCD could serve as an effective drug for the treatment of IgAN. Although the general mechanism underlying the effects of MHCD was revealed, detailed analyses of the precise mechanism will be an important focus of our future research. Specifically, our future work will focus on studying the signaling pathways related to podocyte-associated proteins and the target molecules of MHCD.

## Data Availability

The raw data supporting the conclusion of this article will be made available by the authors, without undue reservation.
